# Extracellular ATP activates MAPK and ROS signaling during injury response in the fungus *Trichoderma atroviride*

**DOI:** 10.3389/fpls.2014.00659

**Published:** 2014-11-21

**Authors:** Elizabeth Medina-Castellanos, Edgardo U. Esquivel-Naranjo, Martin Heil, Alfredo Herrera-Estrella

**Affiliations:** ^1^Laboratorio Nacional de Genómica para la Biodeversidad, CINVESTAV-IrapuatoIrapuato, México; ^2^Departamento de Ingeniería Genética, CINVESTAV-IrapuatoIrapuato, México

**Keywords:** injury response, extracellular ATP (eATP), conidiation, reactive oxygen species (ROS), mitogen-activated protein kinase (MAPK), calcium

## Abstract

The response to mechanical damage is crucial for the survival of multicellular organisms, enabling their adaptation to hostile environments. *Trichoderma atroviride*, a filamentous fungus of great importance in the biological control of plant diseases, responds to mechanical damage by activating regenerative processes and asexual reproduction (conidiation). During this response, reactive oxygen species (ROS) are produced by the NADPH oxidase complex. To understand the underlying early signaling events, we evaluated molecules such as extracellular ATP (eATP) and Ca^2+^ that are known to trigger wound-induced responses in plants and animals. Concretely, we investigated the activation of mitogen-activated protein kinase (MAPK) pathways by eATP, Ca^2+^, and ROS. Indeed, application of exogenous ATP and Ca^2+^ triggered conidiation. Furthermore, eATP promoted the Nox1-dependent production of ROS and activated a MAPK pathway. Mutants in the MAPK-encoding genes *tmk1* and *tmk3* were affected in wound-induced conidiation, and phosphorylation of both Tmk1 and Tmk3 was triggered by eATP. We conclude that in this fungus, eATP acts as a damage-associated molecular pattern (DAMP). Our data indicate the existence of an eATP receptor and suggest that in fungi, eATP triggers pathways that converge to regulate asexual reproduction genes that are required for injury-induced conidiation. By contrast, Ca^2+^ is more likely to act as a downstream second messenger. The early steps of mechanical damage response in *T. atroviride* share conserved elements with those known from plants and animals.

## INTRODUCTION

Wound response is a crucial process for the survival of multicellular organisms and facilitates their adaptation to hostile environments. Plants, being sessile organisms, cannot escape from attack by insects or larger herbivores. Animals, although motile, are also exposed to mechanical damage and injuries inflicted by predators. Similarly, due to their absorptive nutrition mode and their immobility, multicellular (filamentous) fungi are prey to a variety of animal predators including fungivorous nematodes and insects. Nevertheless, the physiological response of fungi to wounding and its implications, if any, remains mostly unexplored.

The topic of damage signals and their perception in different organisms has been of recent interest since key mechanisms such as wound sealing and healing of the damaged tissue, as well as local or systemic responses to prevent infection, contain conserved elements. In highly regenerative animals, wounding can trigger regrowth of a missing body part, involving gene expression changes specific for tissue regeneration (Nacu and Tanaka, 2011; Lee and Gardiner, 2012; Wenemoser et al., 2012). Similarly, plant and moss cells can be reprogrammed to initiate tip growth in wounded tissues (Ishikawa et al., 2011). Unfortunately, our current knowledge of wound response in filamentous fungi is mostly limited to the well-characterized sealing of septal pores by Woronin bodies. This sealing reduces loss of cytoplasmic content to prevent cell death, and is followed by the formation of one or more hyphal tips at the plugged septum, resulting in reinitiation of growth and hyphal reconnection (Jedd, 2011). Despite our limited understanding of wound response in fungi, mechanical damage has been found to trigger entry into development in several species. One of the first reports refers to the formation of fruiting bodies in *Schizophyllum commune* in response to mycelial injury (Leonard and Dick, 1968). These authors suggested the participation of oxidative stress in the production of fruiting bodies in response to injury (Leonard and Dick, 1968). Later, Georgiou et al. (2006) reported the formation of sclerotia in response to oxidative stress in *Sclerotium rolfsii*, and increased reactive oxygen species (ROS) were found in damaged hyphae of the fungus *Glomus intraradices* (Fester and Hause, 2005).

Unfortunately, none of the mentioned reports provided mechanistic insights such that injury-signaling remains poorly understood. In other systems, reliable signals of tissue disruption are known to comprise fragments of the extracellular matrix, extracellular molecules such as ATP, adenosine, RNA or DNA, and certain proteins or protein fragments (Chen and Nuñez, 2010; Zeiser et al., 2011; Heil, 2012). These warning signals are known as damage-associated molecular patterns (DAMPs; Heil and Land, 2014).

The release of ATP is an important early danger signal in humans (Chen and Nuñez, 2010; Zeiser et al., 2011), fish (Kawate et al., 2009), algae (Torres et al., 2008), and plants (Chivasa et al., 2009; Heil et al., 2012). In humans, perception of extracellular ATP (eATP) by purinergic receptors is one of the main biological mechanisms responsible for epithelial intracellular calcium mobilization (Sherwood et al., 2011; Kurashima et al., 2012). In zebrafish, ATP released after an injury is sensed by a purinergic P2Y receptor, which in turn modulates NADPH-oxidase activity (Dual oxidase Duox1; de Oliveira et al., 2014). G-protein-coupled P2Y receptors can also recognize other nucleotides, such as adenosine diphosphate (ADP) and uridine triphosphate (UTP; Abbracchio et al., 2006; Covian-Nares et al., 2010). Purinergic receptors similar to those described in animal systems have not been found in plants. However, an extracellular lectin ATP receptor kinase (LecRK-I.9) was recently discovered in *Arabidopsis* (Choi et al., 2014).

One of the earliest events upon detection of a stress signal is the activation of mitogen-activated protein kinases (MAPKs). MAPKs are part of well-conserved eukaryotic signaling cascades. In *Arabidopsis* at least two MAPKs, MPK6, and MPK3, are rapidly activated by wounding and during plant-pathogen interactions. MPK6 directly phosphorylates the 1-aminocyclopropane-1-carboxylic acid synthases (ACS), ACS6, and ACS2 and thus stabilizes these enzymes, increasing the production of ethylene; while MPK3 activity increases after wounding (Ichimura et al., 2000; Liu and Zhang, 2004; Wang et al., 2008). In *Nicotiana* species, orthologs of MPK6 (SIPK) and MPK3 (WIPK) are activated by wounding (reviewed in Hettenhausen et al., 2014). Plants carrying a mutation in LecRK-I.9 that do not respond to ATP (DORN1), fail to trigger phosphorylation of MPK3 and MPK6 (Choi et al., 2014). Further, plant leaf extracts (which contain multiple DAMPs) can induce secretion of extrafloral nectar, an indirect defense response to damage (Heil et al., 2012).

Based on these precedents we hypothesized that fungi could regulate wound response via MAPK pathways, triggering the activation of developmental processes such as cell growth and differentiation. In most filamentous fungi, there are three MAPK pathways that are involved in several processes, such as asexual and sexual reproduction, general stress response, spore germination, cell fusion, secondary metabolism, and mycoparasitism (Mendoza-Mendoza et al., 2003; Delgado-Jarana et al., 2006; Reithner et al., 2007; Fleissner et al., 2009; Kumar et al., 2010; Lara-Rojas et al., 2011; Bayram et al., 2012; Lichius et al., 2012). Defective sexual and asexual development resulting from MAP kinase mutations have been reported in *Magnaporthe grisea* (Xu et al., 1998), *Fusarium graminearum* (Hou et al., 2002), *Neurospora crassa* (Lichius et al., 2012), and *Aspergillus nidulans* (Wei et al., 2003).

*Trichoderma* species are often a predominant component of the mycoflora in native and agricultural soils, and are considered effective biocontrol agents due to their ability to parasitize phytopathogenic fungi (Herrera-Estrella and Chet, 2003). These ascomycete fungi reproduce asexually forming conidia, as a mechanism for survival and dispersal. Accordingly, the switch from vegetative to reproductive growth is triggered by several types of stress such as light, nutrient deprivation, and acid environments (for a review see Carreras-Villasenor et al., 2012). Recently, Hernández-Oñate et al. (2012) showed that *Trichoderma atroviride* responds to mechanical damage with a morphogenetic change initiated by the activation of regenerative processes, and entry into asexual reproduction (conidiation). Their transcriptome analysis revealed over nine hundred injury-responsive genes, including subsets involved in cell cycle control, oxidative stress, and calcium signaling and transport. The injury response depended on activation of the NADPH-oxidase complex formed by the catalytic (Nox1) and the regulatory (NoxR) subunits, which resulted in ROS production at the hyphal tip within the first minutes after injury. Both Nox1 and NoxR are essential for conidiation since Δ*nox1* and Δ*noxR* mutants do not conidiate in response to this stimulus.

*Trichoderma* species have three MAPKs that belong to the so-called mycoparasitism/filamentous growth, cell wall integrity, and osmotic stress response pathways. In *T. atroviride* they are named Tmk1, Tmk2, and Tmk3, and their corresponding orthologs in yeast are Kss1/Fus3, Slt2, and Hog1 (Mendoza-Mendoza et al., 2003; Delgado-Jarana et al., 2006; Reithner et al., 2007; Zeilinger and Omann, 2007). None of the reports on the role of *Trichoderma*’s MAPKs establishes a connection between them and signaling molecules of the injury response.

Here, we show that eATP serves as a cue that signals tissue disruption to as yet intact hyphae (and, thus, acts as a DAMP) and that Ca^2+^ also plays an important role in asexual reproduction triggered by wounding in *T. atroviride*, likely as a downstream second messenger. We further demonstrate that transduction of eATP signaling takes place through the activation of MAPK cascades involving Tmk1 and Tmk3. Activation of Tmk3 depends on this NoxR/Nox1 complex, whereas that of Tmk1 is independent of the complex. By contrast, calcium signaling appears to take place through a MAPK-independent pathway. In conclusion, DAMPs are involved in the fungal wound response and trigger downstream signaling events that are similar to those known in other organisms.

## MATERIALS AND METHODS

### STRAINS AND CULTURE CONDITIONS

*Trichoderma atroviride* IMI 206040 was used as wild type (WT) strain, the mutants Δ*tmk3* and Δ*tmk1* are described below. The Δ*nox1*, Δ*nox2*, and Δ*noxR* have been reported earlier (Hernández-Oñate et al., 2012). All strains were propagated on potato dextrose agar (PDA) or potato dextrose broth (PDB; Difco) in the dark at 27°C.

### GENERATION OF Δ*tmk1* AND Δ*tmk3* MUTANTS

The open reading frames (ORF) of genes *tmk1* and *tmk3* were replaced by a hygromycin resistance cassette (hph), using the double-joint PCR method, as previously described (Yu et al., 2004). All transformants were subjected to three to five rounds of single spore isolation and gene replacement events verified by PCR and Southern blot (data not shown).

### SOUTHERN BLOT ANALYSIS

The Δ*tmk1* and Δ*tmk3* mutants were verified by Southern blot, following standard procedures (Sambrook and Russell, 2001). Genomic DNA of Δ*tmk1* and Δ*tmk3* was extracted and digested with *Pvu*II and *Eco*RI, respectively, then separated by electrophoresis in a 1% agarose gel, and transferred onto Hybond-N^+^ membranes (Amersham). The probes used to verify the Δ*tmk1* and Δ*tmk3* mutants (2.9 and 3.3 kb) included the complete ORF and 1.5 kb at the 5′ UTR. The probes were labeled with [α^32^P] dCTP by random priming, using the Readyprime kit (Amersham) according to the manufacturer’s specifications.

### INJURY-INDUCED CONIDIATION ASSAYS

All strains tested were grown for 40 h in the dark on a cellophane sheet placed over a single layer of Whatman 1 filter paper. To test the influence of calcium on the response to injury, the strains were exposed to 15 mM ethylene glycol tetraacetic acid (EGTA) (Sigma) for 15 min, or treated with 2 units of apyrase to test the influence of ATP. Mycelia was then damaged with a scalpel or a cookie mold and transferred to fresh media (PBD) and incubated for an additional 48 h in the dark. Subsequently conidia were collected in sterile water and quantified by direct counting in a Neubauer chamber. Strains grown under the same conditions but without treatment were used as controls.

### ATP-INDUCED CONIDIATION ASSAYS

The WT strain was grown in the dark on a cellophane sheet placed on a single layer of Whatman 1 filter for 40 h. The fungus was then transferred to plates containing ATP, ATPγ-S, ADP, CTP, UTP or GTP at a 0.1 mM concentration (Sigma). Additionally, colonies were transferred to plates containing 2 units of apyrase and ATP (0.1 mM) or EGTA (15 mM; Sigma) or *N*-acetylcysteine (NAC; 60 mM) for 15 min. Finally, colonies were washed with sterile water and transferred to Petri dishes containing fresh PDB medium and incubated for additional 48 h in the dark. Control colonies did not receive any treatment. Colonies were photographed; conidia collected in sterile water, and quantified using a Neubauer chamber.

### WESTERN BLOT ANALYSIS

Fresh mycelia with cellophane paper were frozen in liquid nitrogen, ground in a mortar and resuspended in Laemmli’s SDS/DTT sample buffer without dye and maintained on ice. Samples were further disrupted by vortexing; cell and cellophane debris were removed by centrifugation (12000 rpm) for 2 min at 4°C. Protein concentration was determined by using the Bradford assay (Bio-Rad) with BSA as a standard. Equivalent amounts of protein (40 μg) were used for immunoblotting and analyzed by 12% SDS-PAGE followed by electro-blotting onto polyvinyl difluoride (PVDF; Inmobilon®-P) membranes (Milipore, Billerica, MA, USA). The membrane was blocked with 5% low fat milk in TBS-Tween, and probed (overnight at 4°C) with Phospho-p38 MAP Kinase (Thr180/Tyr182) rabbit monoclonal antibody or Hog1 (y-215) polyclonal antibody (Santa Cruz Biotechnology, Santa Cruz, CA, USA) to detect phosphorylated and total Tmk3. Phospho-p42/p44 MAP Kinase (Thr202/Tyr204) polyclonal antibody or p42/p44 MAP Kinase rabbit monoclonal antibodies (Cell Signaling Technology, Beverly, MA, USA) were used to detect phosphorylated and total Tmk1, as indicated. For immunodetection of Tmk-P and total Tmk two separated gels were run using the same sample, transferred onto PVDF membranes and each blot probed with the indicated antibody. Horse Radish Peroxidase-conjugated secondary antibodies (Promega, Madison, WI, USA) and Super Signal West Pico Chemiluminescent Substrate (Pierce-search; Thermo Scientific, Rockford, IL, USA) were used for detection.

### ROS DETECTION ASSAYS

Superoxide detection was performed as described by Lara-Ortíz et al. (2003) with slight modifications. The Δ*nox1*, Δ*nox2*, Δ*noxR* mutants, and the WT strain were grown on cellophane and filter paper in plates containing PDB for 40 h. The filter papers with the fungus were washed with sterile water and transferred to plates with or without 0.3 mM nitroblue tetrazolium chloride (NBT; Sigma) aqueous solution, and incubated for 30 min in the dark at 27°C. Samples were photographed under an inverted microscope.

### STATISTICAL ANALYSIS

The program Graphpad Prism version 6 was used for statistic analysis and constructing graphs. All error bars indicate SEM. A one-way ANOVA test followed by Bonferroni multiple-test correction was used.

## RESULTS

### eATP AND CALCIUM SIGNALING MODULATE CONIDIATION IN RESPONSE TO INJURY

**FIGURE 1:**
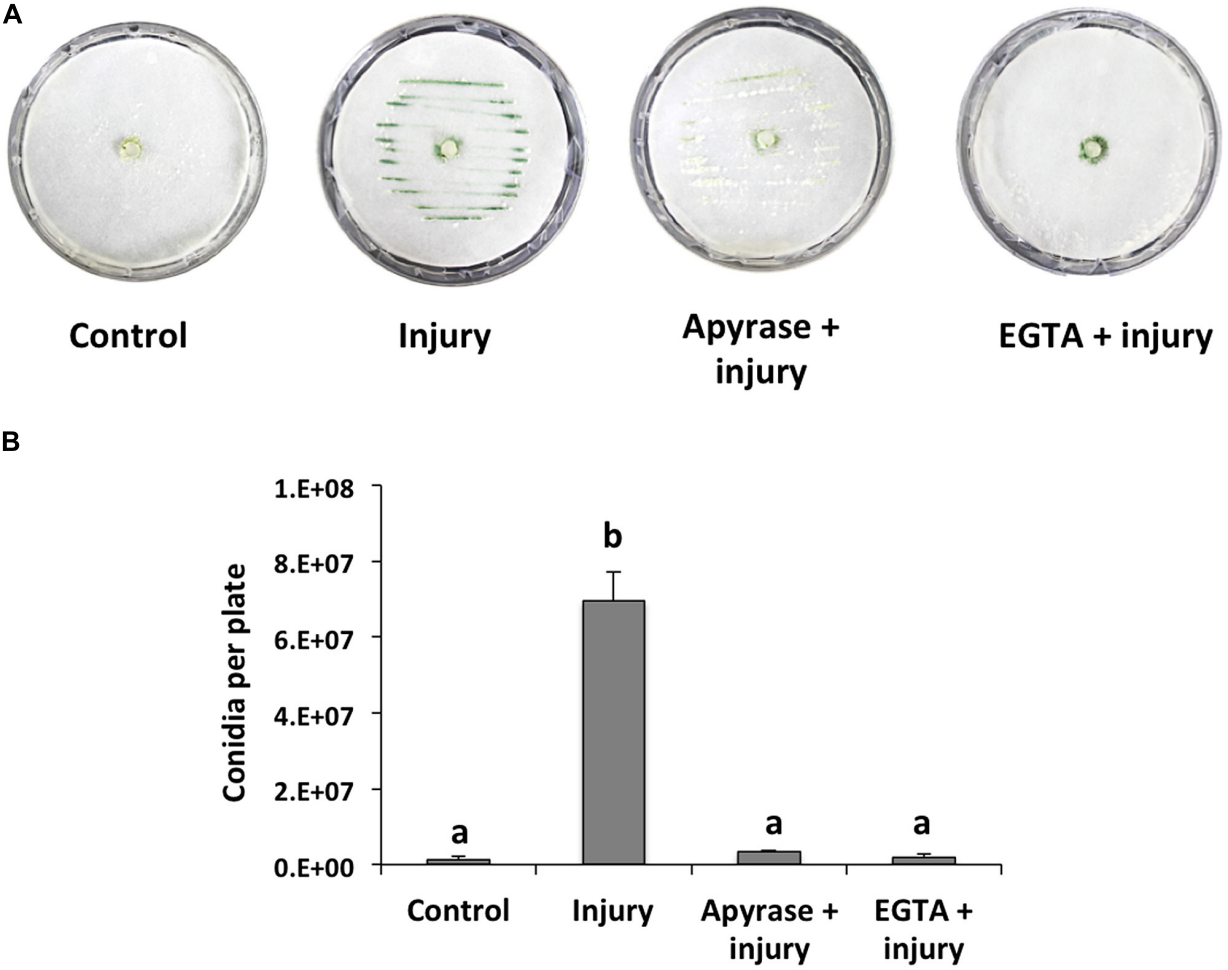
**Effect of EGTA and apyrase on injury-induced conidiation. (A)** Colonies of the fungus were damaged using a scalpel to induce conidiation (visualized as green lines). Prior to damage, apyrase or 15mM EGTA were added. Photographs were taken 48 hours after injury. An undamaged colony is shown as control. **(B)** Quantification of conidia produced after injury. Error bars represent the mean ± SEM of three biological replicas. Bars with different letters indicate treatments that were significantly different (*P* < 0.001).

When a hypha is damaged, release of cytoplasmic content is inevitable and, thus, surrounding healthy cells could recognize its components as a danger signal. Accordingly, we first focused on extracellular signals that might be responsible for conidiation during the wound response in *T. atroviride*, and evaluated eATP and Ca^2+^ as potential damage signal molecules. For this purpose, we incubated *T. atroviride* with apyrase, an enzyme that hydrolyzes eATP, or an extracellular Ca^2+^ chelating agent. Degradation of ATP by apyrase or trapping Ca^2+^ with EGTA in colonies that were damaged with a scalpel resulted in strongly reduced conidiation in the wound region (96 and 98%, respectively), as compared with an injured control (**Figures 1A,B**). These observations suggested that eATP and Ca^2+^ play a major role in the wound response.

**FIGURE 2:**
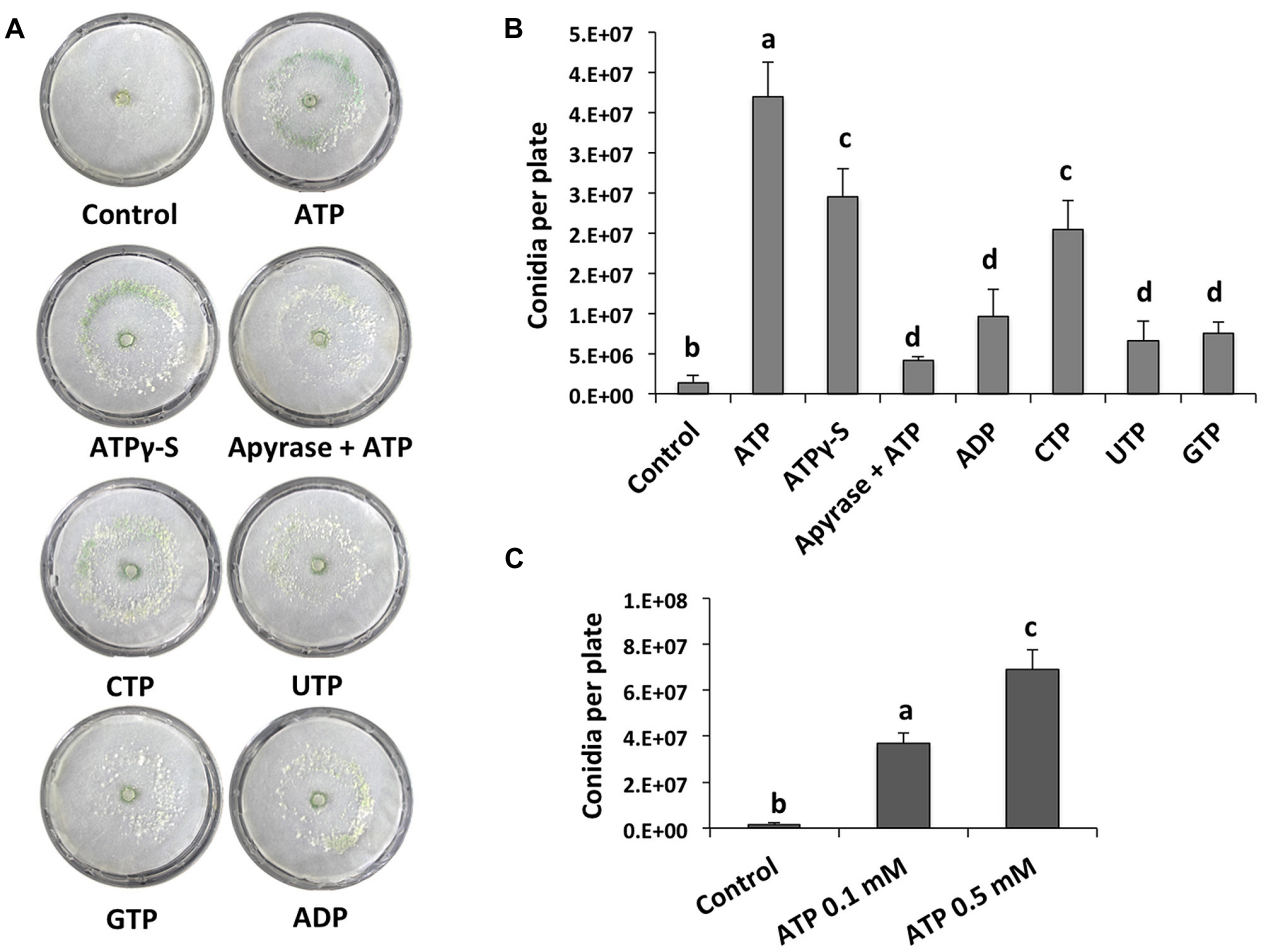
**eATP stimulates conidiation. (A)** Analysis of the WT strain in response to ATP, ATPγS, ADP, CTP, UTP, and GTP (0.1 mM), or ATP and 2 units of apyrase. Photographs were taken 48 hours after treatment. An undamaged colony is shown as control. **(B)** Quantification of conidia produced after the treatments shown in **(A)**. **(C)** Quantification of conidia produced in response to different ATP concentrations. Error bars represent the mean ± SEM of three biological replicates. Bars with different letters indicate treatments that were significantly different (*P* < 0.01).

We analyzed the effect of adding eATP and found that it strongly induced conidiation in the peripheral region of an undamaged colony (**Figure 2A**). We also tested the effect of different purine and pyrimidine triphosphate compounds, ADP, and ATPγ-S (a non-hydrolysable analog of ATP). Conidiation was induced by CTP and ATPγ-S, although not to the same extent observed upon application of ATP (**Figures 2A,B**). The purine nucleotides ADP and guanosine triphosphate (GTP), as well as the pyrimidine nucleotide UTP had only a minor impact on the production of conidia (**Figures 2A,B**). These data suggest that energy derived from ATP hydrolysis is not required for the induction of conidiation, and that a putative receptor with higher affinity for ATP than for other nucleotides is required. Finally, conidiation induction by eATP appears to be dose-dependent (**Figure 2C**).

### Tmk1 AND Tmk3 ARE ACTIVATED IN RESPONSE TO INJURY AND eATP

**FIGURE 3:**
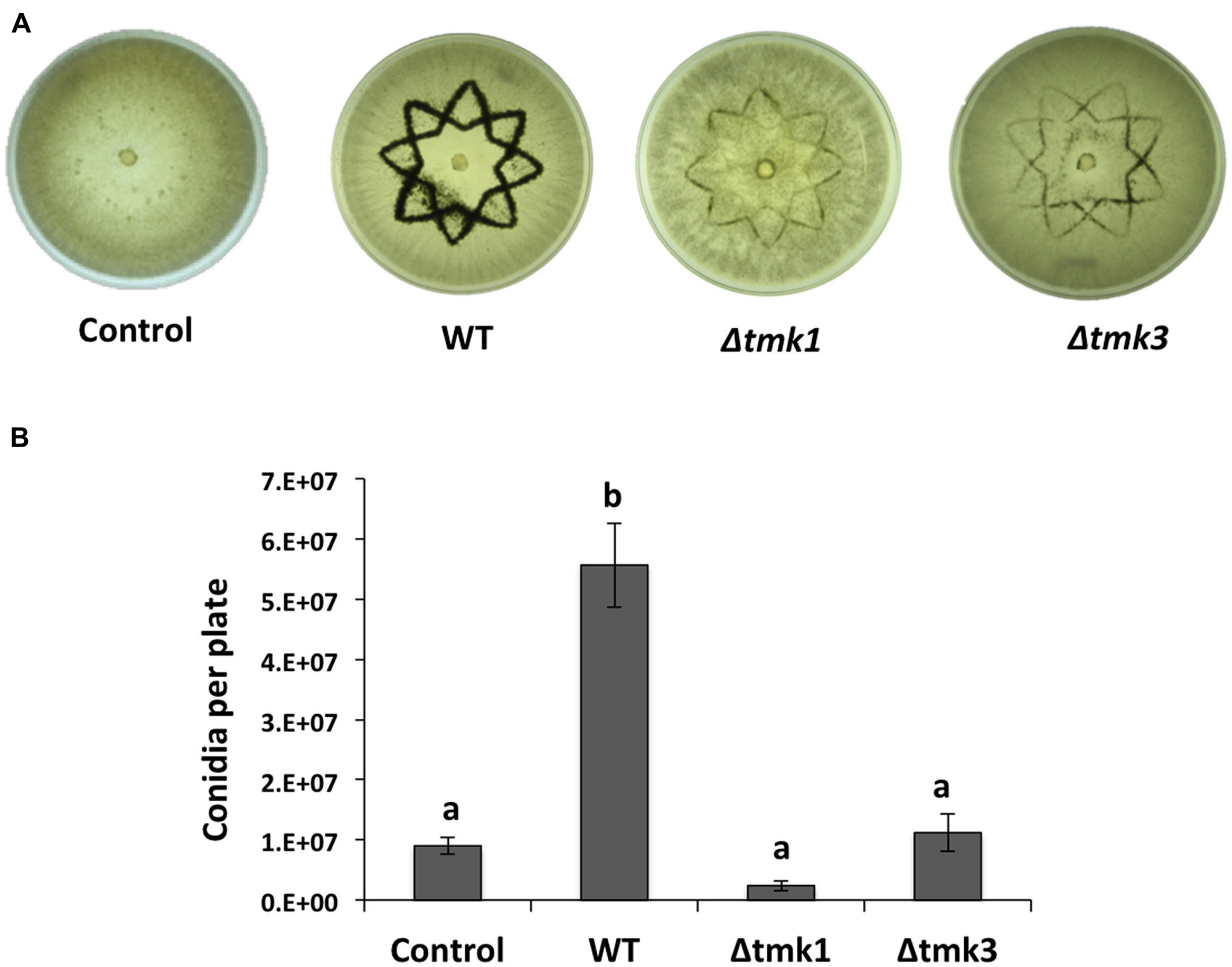
**Injury response of the Tmk1 and Tmk3 mutants. (A)** The WT, Δ*tmk1* and Δ*tmk3* strains growing on PDA were damaged with a cookie mold, and photographs taken 48 hours later. An undamaged WT strain is shown as control. (B) Quantification of conidia produced after injury for each strain. Error bars represent the mean ± SEM of three biological replicas. Bars with different letters indicate treatments that were significantly different (P < 0.001).

To determine if a MAPK pathway is involved during wound response, we evaluated mycelial growth and conidiation in the knockout mutants Δ*tmk1* and Δ*tmk3* in response to injury. We damaged hyphae of WT, Δ*tmk1* and Δ*tmk3* strains with a star shaped cookie mold and observed that the Δ*tmk1* strain did not produce aerial mycelia in response to damage, whereas the Δ*tmk3* and WT produced aerial mycelia to a similar extent (data not shown). Both Δ*tmk1* and Δ*tmk3* mutants exhibited a dramatic decrease in conidia production (**Figure 3A**), with reductions of 95 and 80%, respectively (**Figure 3B**). This suggests that transduction of injury related signals leading to conidiation is modulated mainly by MAPK pathways.

**FIGURE 4:**
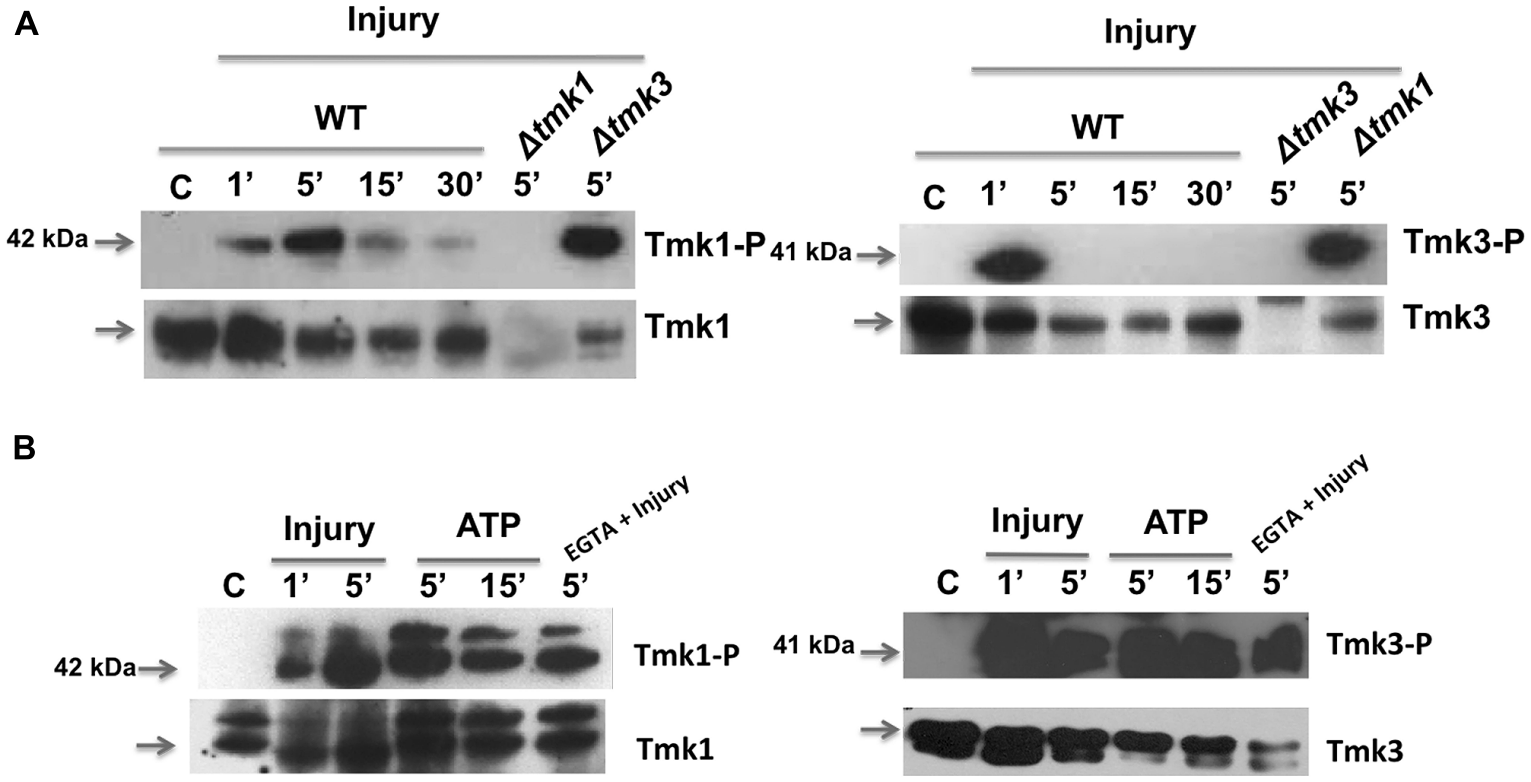
**Phosphorylation of TMK1 and TMK3 in response to injury and eATP. (A)** The WT strain was injured and mycelial samples collected at the indicated times. Mycelium from an undamaged colony was included as control (C). Proteins were extracted, separated by SDS-PAGE, and used for immunoblotting. Blots were probed with anti-Tmk1 (anti-p42/p44) and Tmk1-P (anti-Phospho-p42/p44) antibodies (left panel) or anti-Tmk3 (antip38) and Tmk3-P (anti-Phospho-p38) antibodies (right panel). Note that the anti-Tmk1 antibodies also recognize Tmk2 (p44), as previously shown (Mendoza-Mendoza et al., 2003). **(B)** The WT strain was ATP induced (0.1 mM) or treated with EGTA (15 mM) and mycelial samples collected at the indicated times. Proteins were extracted, separated by SDS-PAGE, and used for immunoblotting. Blots were probed as in **(A)**. Arrows indicate the bands corresponding to Tmk1 or Tmk3. The Δ*tmk1* and Δ*tmk3* mutants were included as controls. The experiments were repeated three times with similar results.

To further investigate the activation of MAPK pathways by wounding and eATP, we performed western blots using specific antibodies to detect phosphorylation of Tmk1 and Tmk3. Tmk1 was phosphorylated very rapidly, within the first five minutes after wounding, to then decrease but still slightly detectable up to 30 min later; whereas Tmk3 exhibited maximum phosphorylation a minute after injury, decreasing very rapidly afterwards (**Figure 4A**). This suggests that Tmk1 plays a sustained role, while Tmk3 participates only during the immediate response.

Given that injury activates MAPK pathways and that ATP is essential for, and mimics this response, we tested if eATP is sufficient to activate MAPK pathways. Application of eATP to *T. atroviride* activated both MAPK pathways following similar kinetics to those observed after injury (**Figure 4B**). Using the Tmk1-Phospho and total antibodies we observed two bands for Tmk1 identification, due to the fact that the antibodies recognize two MAPKs; Tmk1 (p42) and Tmk2 (p44). However, the antibodies used showed higher affinity for Tmk1, as shown in **Figure 4B**. This result indicates that both stimuli use the Tmk1 and Tmk3 pathways for signaling. Tmk1 and Tmk3 were phosphorylated even when extracellular calcium was chelated by added EGTA (**Figure 4B**; EGTA + injury). Together these results suggest that there are at least three signaling pathways involved in the wound response, two of them regulated by MAPKs and the third one involving calcium signaling.

### eATP SIGNALING PROMOTES Nox1-DEPENDENT ROS PRODUCTION AND REQUIRES ACTIVATION OF A MAPK PATHWAY

**FIGURE 5:**
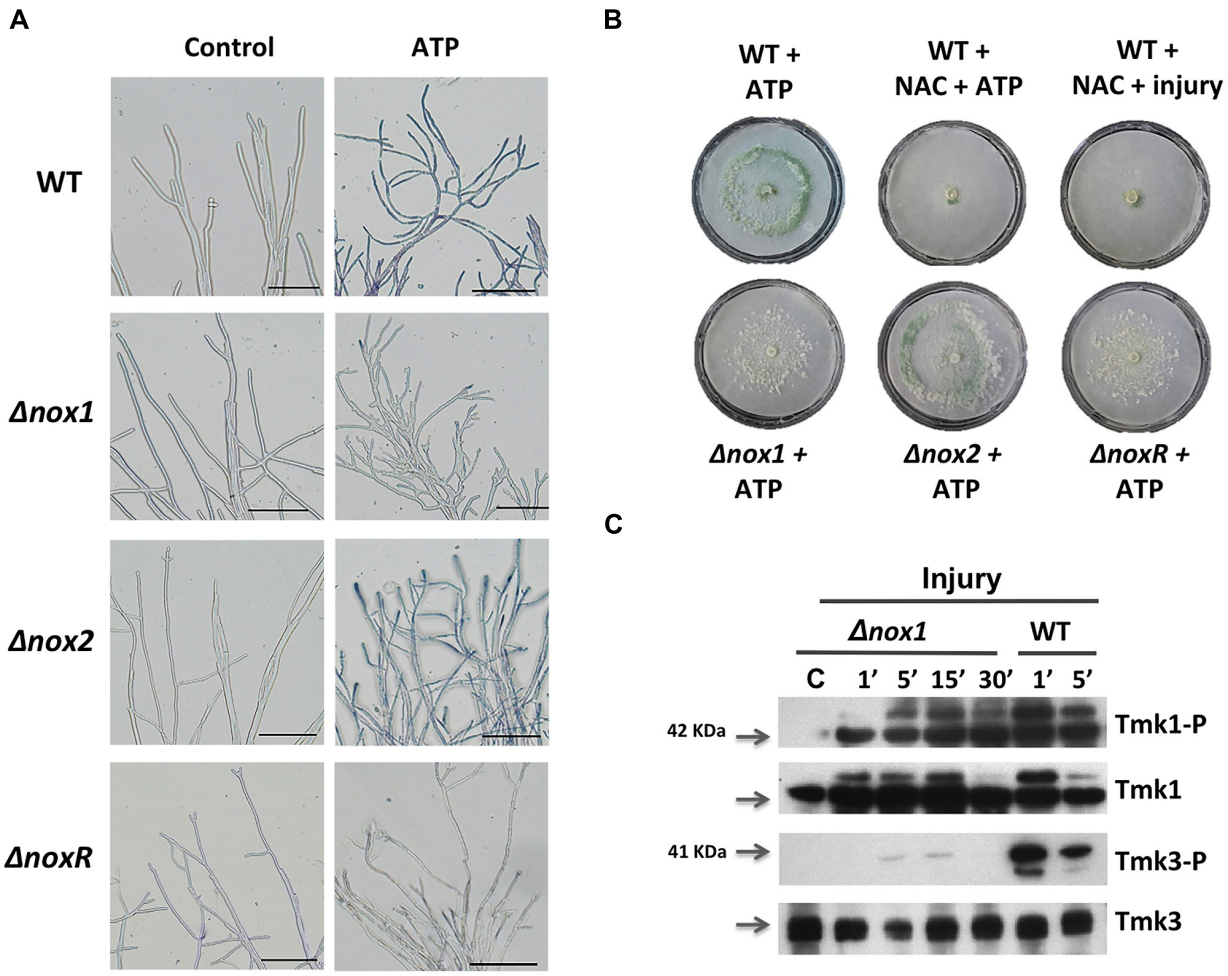
**Production of superoxide in response to extracellular ATP (eATP). (A)** Detection of superoxide. WT, Δ*nox1*, Δ*nox2*, and Δ*noxR* strains were incubated with ATP (0.1 mM), followed by incubation in a 0.3 mM NBT solution and examined by bright-field microscopy (BF). The blue/purple coloration indicates the production of superoxide (formazan generation). The scale bar = 10 μm. **(B)** eATP-induced conidiation. The WT strain was treated with ATP (0.1 mM), or a combination of NAC (60 mM) and ATP, or NAC and injured with a scalpel. The Δ*nox1*, Δ*nox2*, and Δ*noxR* mutants were induced with ATP (0.1 mM). **(C)** The Δ*nox1* mutant was injured and mycelial samples collected at the indicated times. Proteins were extracted, separated by SDS-PAGE, and used for immunoblotting with anti-Tmk1 (anti-p42/p44), Tmk1-P (anti-Phospho-p42/p44), anti-Tmk3 (antip38) and Tmk3-P (anti-Phospho-p38) antibodies. Mycelium from an undamaged colony was included as control (C). Arrows indicate the bands corresponding to Tmk1 or Tmk3. The experiments were repeated two times with similar results.

Injury-stimulated Nox1-dependent ROS production is essential for conidiation (Hernández-Oñate et al., 2012). To determine whether eATP could activate NADPH oxidase (Nox1/NoxR)-dependent ROS production, triggering conidiation, we analyzed the production of superoxide and conidia in response to eATP in the Δ*nox1*, Δ*nox2*, and Δ*noxR* mutants. Samples of mycelia collected 15 min after eATP induction in the presence of Nitroblue tetrazolium chloride (NBT) were used to detect production of superoxide. After a few minutes of eATP exposure, hyphal tips of the WT and Δ*nox2* strains showed the characteristic dark-blue precipitate indicating formazan formation. In contrast, the Δ*nox1* and Δ*noxR* strains failed to produce superoxide at the hyphal tips (**Figure 5A**). To test whether eATP promotes conidiation in the absence of Nox1-dependent ROS, we exposed the WT strain to eATP or injured it in the presence of the antioxidant N-acetyl cysteine (NAC). In both cases conidiation was abolished (**Figure 5B**). Similarly, the Δ*nox1* and Δ*noxR* mutants did not conidiate in response to eATP. In contrast, the Δ*nox2* mutant strain conidiated similarly to the WT (**Figure 5B**). These observations indicate that eATP stimulates Nox1-dependent ROS production (acts downstream of eATP); hence we decided to explore the activation of Tmk1 and Tmk3 in the Δ*nox1* mutant. Tmk1 activation appeared to be Nox1 independent, since it was still phosphorylated after injury in the absence of Nox1, while Tmk3 phosphorylation was not observed in the Δ*nox1* strain (**Figure 5C**). Together these results strongly suggest that eATP is a cell-damage signal that promotes the production of ROS by Nox1, which in turn activates Tmk3, whereas calcium signaling participates independently of the MAPK pathways.

## DISCUSSION

Wound detection and healing represent important processes for the survival of any multicellular organism, involving conserved mechanisms across species. Multicellular fungi respond to wounding by sealing the septa of the cell adjacent to the damaged one by rapidly mobilizing proteins and forming Woronin bodies (Jedd, 2011). Interestingly, several fungi respond also by initiating a morphogenetic change that leads to the formation of different structures, such as sexual fruiting bodies, and conidiophores (Leonard and Dick, 1968; Hernández-Oñate et al., 2012). Unfortunately, there is scarce information about the perception of potential danger signaling molecules and their transduction. To the best of our knowledge, the present report is the first that contributes to understanding the signaling pathways involved in the wound response in filamentous fungi.

Hernández-Oñate et al. (2012) recently showed that mycelial injury in *T. atroviride* results in the formation of conidiophores that are produced exclusively from the newly regenerated hyphae. This observation suggests that signal molecules are released to the extracellular matrix during injury and that adjacent cells recognize these molecules. In plants, eATP is considered a damage signaling molecule, included in a group of molecules known as DAMPs, produced during herbivory, mechanical damage, or pathogen attack (Roux and Steinebrunner, 2007; Chivasa et al., 2009; Heil, 2012; Heil et al., 2012). Interestingly, our data show that eATP induces conidiation in response to injury and suggest that eATP is an important signal molecule that is released from damaged hyphae. Thus, eATP can also be considered a damage signaling molecule in fungi, which plays a similar role in wound signaling to that reported in triggering immune responses in mammals (Chen and Nuñez, 2010; Zeiser et al., 2011; Cordeiro and Jacinto, 2013), fish (Kawate et al., 2009), insects (Moreno-García et al., 2014), algae (Torres et al., 2008), and plants (Chivasa et al., 2009). Elevations in the concentration of intracellular calcium have also been observed in *Arabidopsis* upon application of eATP (Tanaka et al., 2010). Further, the induction of conidiation by eATP displayed a dose-dependent trend. This observation suggests that the fungus senses the concentration of eATP, which is probably correlated with the level or extension of the injury, and, thus produces more conidia to warrant survival when it suffers from particularly strong damage.

In accordance with reports of plants and animals, where Ca^2+^ influxes into the cytosol follow the perception of DAMPs (Heil and Land, 2014), our observation that chelating extracellular calcium blocked injury-induced conidiation would suggest that calcium plays a key role as second messenger of wounding. The early wound signaling response in animals, including humans (Shabir and Southgate, 2008; Covian-Nares et al., 2010), and plants (Arimura and Maffei, 2010; Beneloujaephajri et al., 2013) includes an increase in intracellular calcium and the activation of the calcium signaling machinery. Interestingly, calcium induces the formation of conidia in submerged cultures of *T. viride* (Simkovic et al., 2008). Furthermore, transcriptomic analysis of the response to injury in *T. atroviride* suggested the participation of a calcium signaling pathway, since several genes related to calcium signaling, including calcium transporters, phospholipase C, and a Ca^2+^/calmodulin-dependent kinase-1 (CAMK-1) were induced (Hernández-Oñate et al., 2012). In agreement with these observations, Nelson et al. (2004) showed that hypo-osmotic shock and external calcium treatment induce transient increase in intracellular calcium in *Neurospora crassa*, *Aspergillus niger*, and *Aspergillus awamori*. Our results strongly indicate that eATP is a damage signal, and that calcium acts downstream of this DAMP.

The recognition of nucleotides through purinergic receptors (a family of receptors initially classified according to the relative potency of purine nucleotides to stimulate them), which exhibit different affinities for different nucleotides, is well known in animal systems. Efforts to identify plant ATP receptors through homology of their genomic sequence to animal purinergic receptors failed to find any suitable candidates, but recently the lectin receptor kinase-I.9 (LecRK-I.9) has been shown to perceive eATP (Choi et al., 2014). In contrast, no single nucleotide receptor has been reported in fungi to date. However, our results indicate that there must be a nucleotide receptor, with higher affinity for ATP than for other nucleotides.

Here we show that *T. atroviride* also responds to extracellular CTP by forming conidia, consistent with evidence in *Arabidopsis*, where a significant elevation in cytosolic Ca^2+^ could be elicited by the application of exogenous ATP or CTP but not by ITP, TTP, or UTP ( Tanaka et al., 2010; Choi et al., 2014). In addition GTP, CTP, and UTP (as well as ATP) were found to induce superoxide production in *Arabidopsis* leaves (Song et al., 2006).

Although a BLAST based search for homologues of lectin receptor kinases in the *T. atroviride* genome database failed to find any match, the kinase domain of the lectin receptor presents high similarity with a MAPKKK orthologous to yeast Bck1, which participates in the protein kinase C signaling pathway that controls cell wall integrity (Lee and Levin, 1992; Lee et al., 1993). In this sense, one of the earliest signaling events after wound in animals and plants is the activation of MAPKs (Wu and Baldwin, 2010; Suzuki and Mittler, 2012). The first report of the involvement of MAPKs in plant–herbivore interactions showed that transcription and activity of wound-induced protein kinase (WIPK), a member of MAPK subfamily A, increased 1 min after wounding (Seo et al., 1995). In filamentous fungi, MAPKs play a central role in development and sexual/asexual reproduction (Xu et al., 1998; Hou et al., 2002; Wei et al., 2003; Lara-Rojas et al., 2011; Lichius et al., 2012). In agreement, Tvk1, the *T. virens* ortholog of Tmk1, is involved in conidiation and the activation of genes encoding cell wall proteins (Mendoza-Mendoza et al., 2007), and the ortholog of Tmk3 in *T. harzianum* plays an important role in the oxidative and osmotic stress response (Delgado-Jarana et al., 2006). Nevertheless, according to these reports there was no evidence of their involvement during wound response.

Here we describe, for the first time in a filamentous fungus, the activation of MAPKs upon wounding, as well as the phosphorylation of two of them (Tmk1 and Tmk3) in response to eATP. Interestingly, we observed that Tmk1 protein levels decreased in the *tmk3* mutant background. In this regard, in *Saccharomyces cerevisiae* Hog1 (the ortholog of Tmk3) induces changes in RNA Pol II localization, with a shift toward stress-responsive genes (Nadal-Ribelles et al., 2012). Therefore, it is tempting to speculate that lack of *tmk3* could result in a modified expression profile, including decreased expression of *tmk1*. Remarkably, Tmk2 appears to also be induced by injury and eATP.

During tissue regeneration and healing in *Drosophila melanogaster*, Grainy head, a transcription factor responsible for epidermal barrier formation and repair, is phosphorylated by ERK1 (Rämet et al., 2002; Kim and McGinnis, 2011), and the activation of ERK is required in mammalian cells for both restoration of damaged tubular epithelial cells and inhibition of fibrosis progression following injury (Jang et al., 2013). Consistently, Tmk1, the *T. atroviride* ortholog of mammalian ERK1/2 and plant MAP2K1, as well as Tmk3, the ortholog of mammalian MAPK p38 and plant MAPK3, were also activated by wounding (for a review see Taj et al., 2010). We further showed that eATP induces Nox1-dependent ROS production, and that this activates exclusively the Tmk3 pathway. The Tmk1 pathway is likely activated by small GTPases, as proposed in previous reports (Schmoll, 2008). Similarly, Hernández-Oñate et al. (2012) showed in *T. atroviride* that wounding promotes ROS production through Nox1.

Interestingly, a recent report in the fungus *Ganoderma lucidum* revealed that Nox-generated ROS elevated cytosolic Ca^2+^ levels by activating a plasma membrane Ca^2+^ influx pathway, thereby regulating ganoderic acid biosynthesis and hyphal branching (Mu et al., 2013). ROS play essential roles in sexual development in *Aspergillus nidulans* and *N. crassa* (Lara-Ortíz et al., 2003; Cano-Domínguez et al., 2008), as well as cell signaling roles (Aguirre et al., 2005). In agreement with these observations, we showed that *T. atroviride* responds to eATP by developing asexual structures. In animals, production of ROS by Nox1/NoxR or Dual oxidases is crucial for the inflammatory response or activation of systemic defense after wounding (de Oliveira et al., 2014). In plants and animals, Ca^2+^ stimulates Nox1/NoxR and Dual oxidase activity through their EF-hand calcium-binding domain, a domain not found in fungal Nox1/NoxR. These enzymes in turn produce ROS, which provoke liberation of intracellular Ca^2+^, likely causing feed back regulation (Niethammer et al., 2009; Wu and Baldwin, 2010; Razzell et al., 2013). The precise role of Ca^2+^ in the damage response of *Trichoderma* remains to be proven, since calcium released from a broken cell could be detected by neighboring cells as a signal molecule, but it could also serve as a second messenger liberated from intracellular pools or transported across the plasma membrane upon detection of DAMPs. Our results suggest that eATP induces conidiation in a Ca-independent manner, as phosphorylation of Tmk1 and Tmk3 occurred also in the presence of the Ca-chelating agent, EGTA. On the other hand, increasing the extracellular concentration of Ca^2+^ was sufficient to trigger conidiation. Thus, we hypothesize that more than one signaling pathway may converge in triggering the expression of genes that are required for the wound-induced formation of conidia.

**FIGURE 6:**
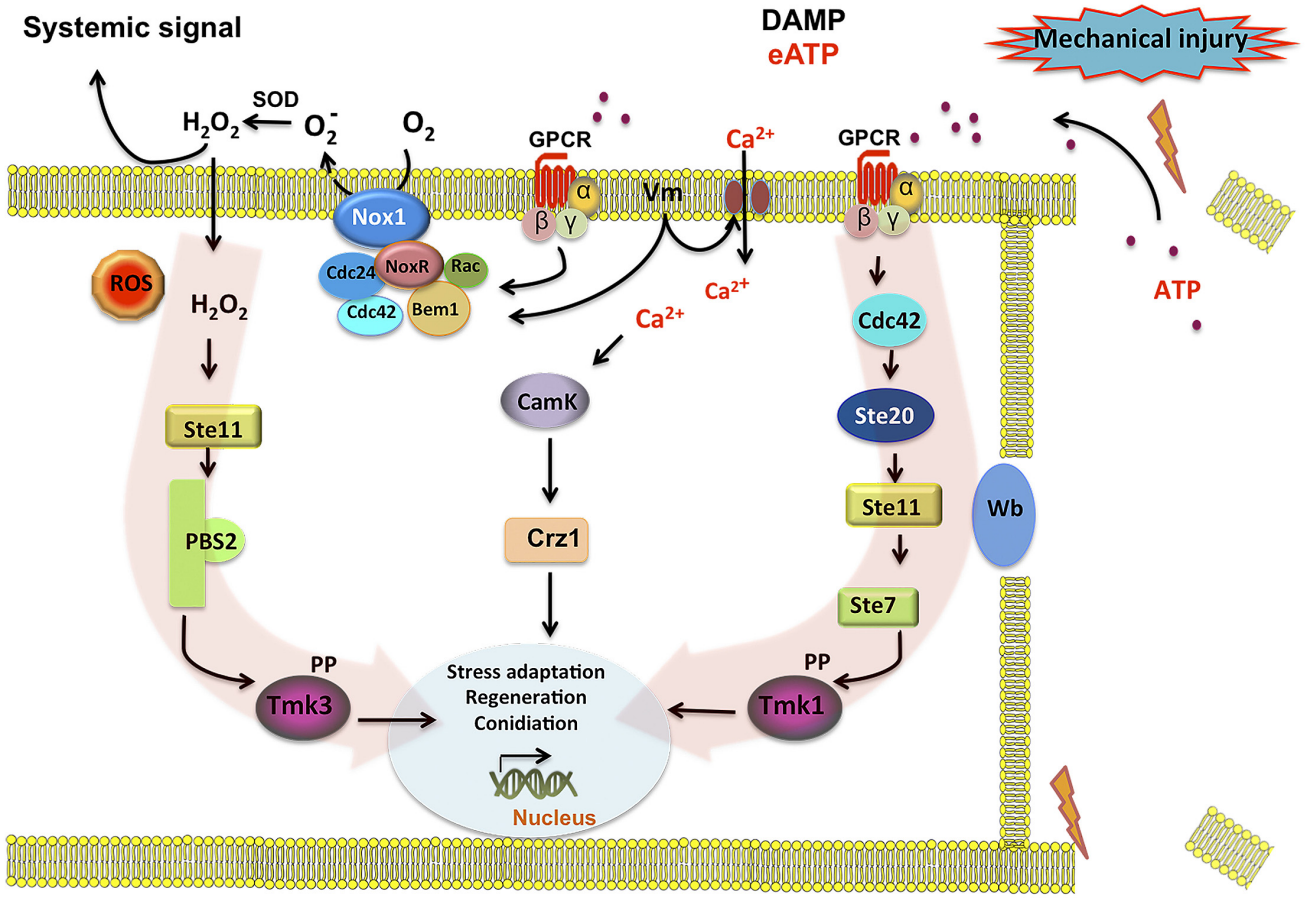
**Model for injury-induced signaling in *Trichoderma atroviride*.** Broken hyphae release ATP as a signal molecule. ATP is perceived by a putative G protein-coupled receptor (GPCR) activating the Tmk1 and Tmk3 MAPK pathways (highlighted by red arrows). Activation of the GPCR turns on the Cdc42 GTPase in coordination with an increase of Nox1-dependent reactive oxygen species (ROS) production. Cdc42 may in turn activate the Ste20 MAPK pathway, leading to Tmk1 phosphorylation. In a parallel pathway increases in intracellular calcium and CamK kinases, regulate targets required for the damage response. Calcium influx may also lead to changes in membrane potential (Vm) and/or directly activate the Rac GTPase component of the NADPH oxidase (Nox1/NoxR) complex, generating $O_{2}^{-}$. A superoxide dismutase (Sod) converts $O_{2}^{-}$ into H_2_O_2_ that can diffuse into the cell, activating the Ste11 MAPK pathway, leading to Tmk3 phosphorylation. Phosphorylation of Tmk1 and/or Tmk3 results in the activation of the developmental program that results in the formation conidia.

In summary, we have shown that eATP can serve as DAMP that activates ROS production by Nox1. Tmk3 is activated in a NoxR–Nox1 dependent fashion, whereas Tmk1 is independently activated. In parallel, Ca^2+^ signaling, which appears to be essential for the wound response, likely activates CAMK or the PKC pathway, to finally trigger a transcriptional response that turns on genes related to cellular stress, regeneration, and conidiation. **Figure 6** shows a model of the cellular response to damage of *T. atroviride*, where we propose eATP as a damage molecule released from wounded hyphae that promotes Nox1-dependent ROS production and in turn activates Tmk3 phosphorylation. Activation of Tmk1 appears to be independent of ROS and extracellular Ca^2+^ but important for injury-induced conidiation. Nevertheless, we can not exclude the possible activation of Tmk1 by Ca^2+^ mobilized from intracellular pools in response to damage. In conclusion, wound signaling in *T. atroviride* is an evolutionarily conserved process displaying multiple similarities to processes described in other higher eukaryotes. The relative simplicity of *T. atroviride* makes it an excellent model for the study of wound response and regeneration in multicellular organisms.

## Conflict of Interest Statement

The authors declare that the research was conducted in the absence of any commercial or financial relationships that could be construed as a potential conflict of interest.
